# What Is Your Diagnosis? Salivary Gland Mass in a Dog

**DOI:** 10.1111/vcp.70091

**Published:** 2026-01-20

**Authors:** Hyeonseung Back, Jihun Won, Taekun Kim, Yongsun Kim, Ulsoo Choi

**Affiliations:** ^1^ Department of Veterinary Clinical Pathology, College of Veterinary Medicine Jeonbuk National University Iksan‐si Jeollabuk‐do Republic of Korea; ^2^ BON Animal Medical Center Suwon‐si Gyeonggi‐do Republic of Korea

**Keywords:** dog, lipomatosis, salivary gland

## 1 Case Presentation

A 10‐year‐old spayed female German Shepherd dog was referred to BON Animal Medical Center for a mass extending from the facial region into the oral cavity. Computed tomography (CT) revealed a large mass with fat to soft tissue density involving the left buccal region, soft palate, zygomatic gland, and between muscle layers (Figure 1). The portion extending toward the soft palate protruded into the nasopharyngeal area, partially narrowing the lumen. Surgical excision included removal of the affected zygomatic gland. The excised mass was subjected to fine‐needle aspiration and cytologic examination at the clinic (Figure 2).

**FIGURE 1:**
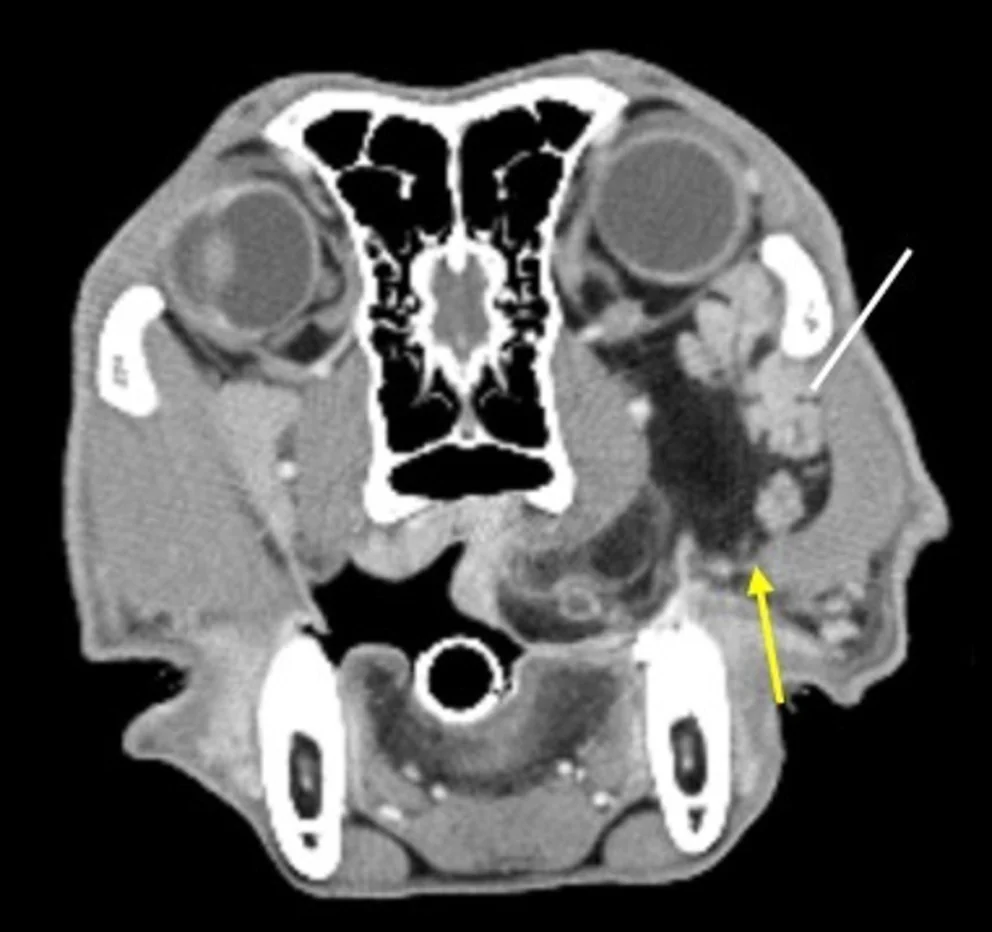
Transverse plane computed tomography (CT) image of the head of a 10‐year‐old German Shepherd dog. A large, tumor‐like mass with fat‐to‐soft‐tissue density involves the zygomatic gland. Yellow arrow: Mass lesion, White line: Zygomatic gland.

**FIGURE 2:**
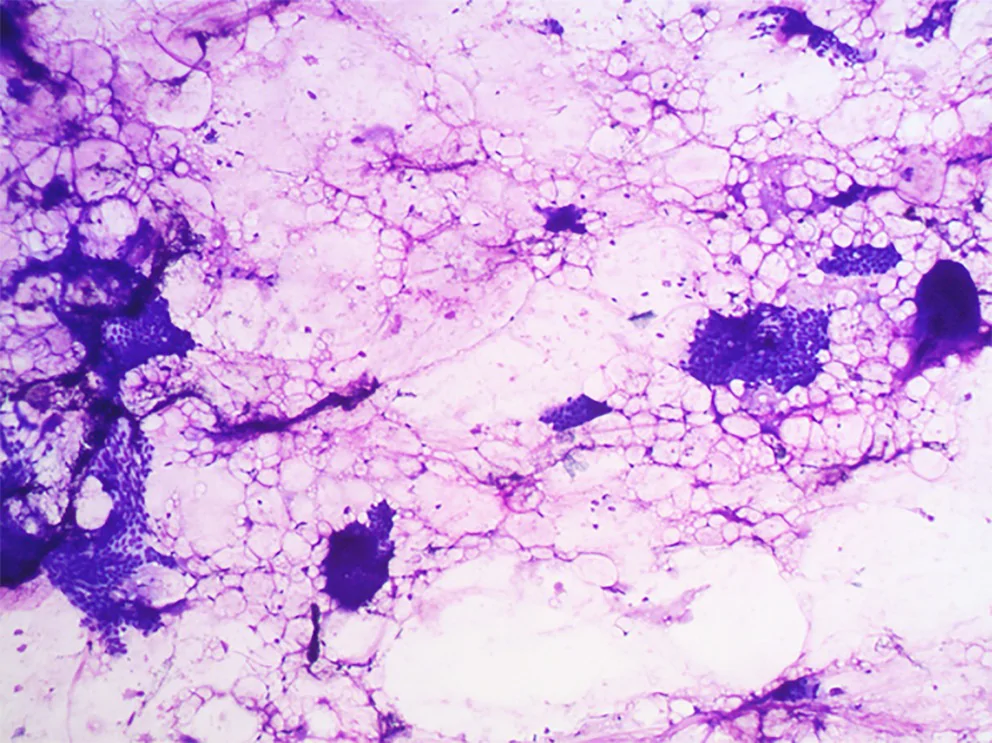
Cytologic photomicrograph of a zygomatic salivary gland mass in a 10‐year‐old German Shepherd dog. Well‐differentiated adipocytes are intermixed with salivary gland epithelial cell clusters. Cellular atypia is not observed. Aqueous Romanowsky stain, ×20 objective.

### 1.1 Cytologic Interpretation

Salivary gland lipomatosis.

On cytologic examination, the specimen was moderately cellular. It consisted of well‐differentiated adipocytes mixed with a low number of secretory or ductal epithelial cell clusters, compatible with salivary gland tissue. The background contained eosinophilic to lightly basophilic mucus and numerous lipid droplets. Adipocytes exhibited a low nucleocytoplasmic ratio, with clear cytoplasm largely occupied by lipid vacuoles. Nuclei were round to oval, paracentral in location, with condensed chromatin and inconspicuous nucleoli. Anisocytosis and anisokaryosis were mild. Epithelial cell clusters exhibited a low to moderate nucleocytoplasmic ratio, with pale basophilic, foamy cytoplasm. Nuclei were round to oval, with condensed chromatin and inconspicuous nucleoli. Anisocytosis and anisokaryosis were mild to moderate. Some epithelial cells exfoliated as cohesive sheets with a higher nucleocytoplasmic ratio and fewer cytoplasmic vacuoles than other epithelial cells. There was no evidence of inflammation or infectious agents. Based on the lesion description and imaging findings, the cytological features were suggestive of salivary gland lipomatosis. Other differential diagnoses included infiltrative lipoma and salivary gland epithelial hyperplasia with inadvertent aspiration of subcutaneous fat.

## 2 Additional Results

The mass was surgically removed, fixed in 10% neutral‐buffered formalin, and submitted to a commercial laboratory for evaluation. Histopathology showed multiple hypercellular lobules composed of well‐differentiated adipocytes that disrupted and separated salivary gland lobes (Figure 3). Salivary gland lobes were sometimes atrophic and surrounded by fibroplasia, and no cellular atypia or mitotic activity was observed. Histologic margins consisted of normal adipose tissues. These findings were consistent with salivary gland lipomatosis. The patient recovered uneventfully from surgery and remained clinically healthy without any signs of recurrence or complications.

**FIGURE 3:**
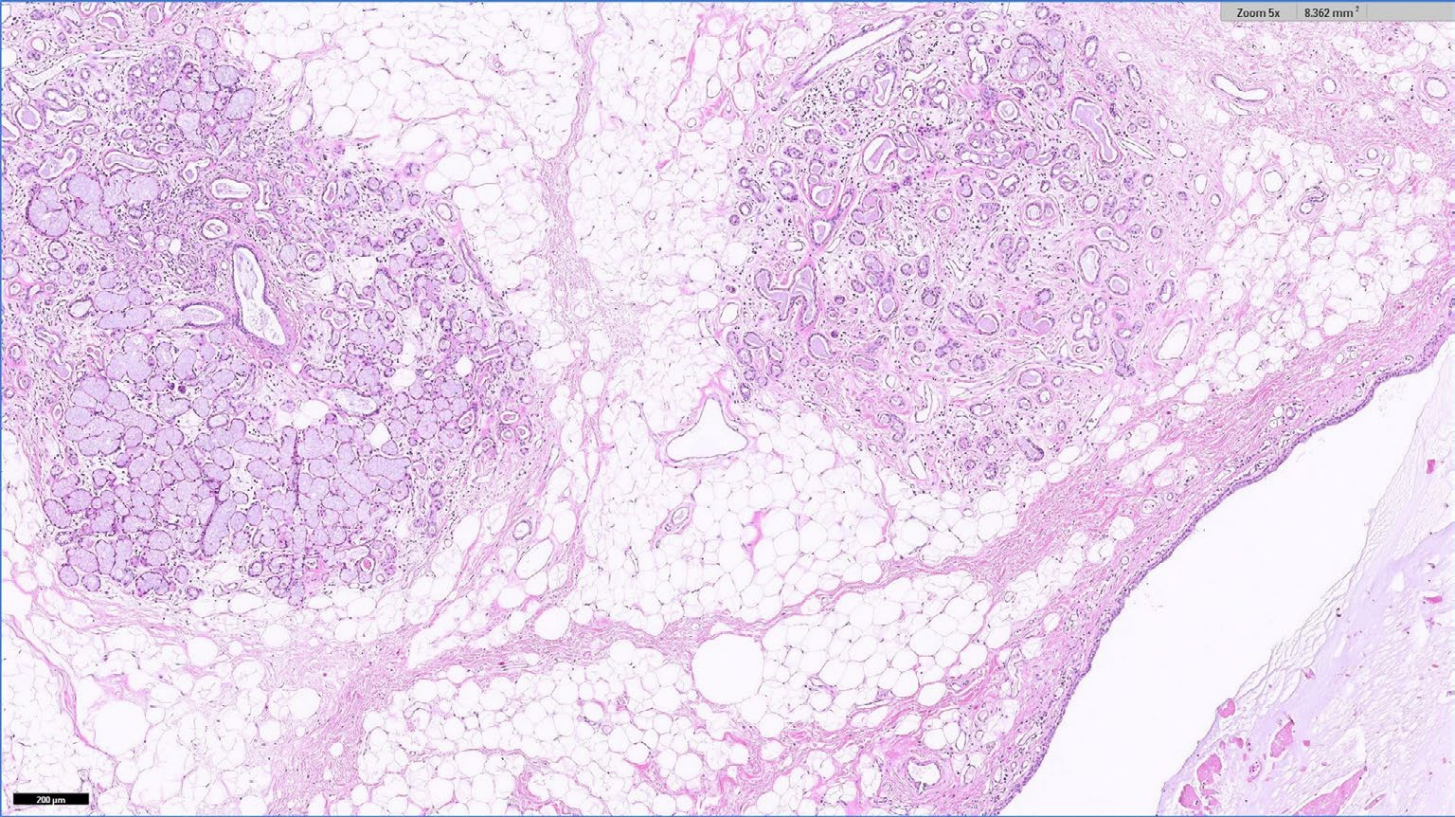
Histologic sections of a zygomatic salivary gland mass in a 10‐year‐old German Shepherd dog. Salivary gland lobes are separated by multiple hypercellular lobules composed of well‐differentiated adipocytes. Hematoxylin and eosin stain, ×5 objective.

## 3 Discussion

Salivary gland lipomatosis, which could be considered a benign‐like tumor‐like lesion with an incompletely understood pathophysiology, is a rare condition in dogs. Several hypotheses have been proposed, suggesting that the condition may be secondary to an underlying obstructive or inflammatory disease of the gland [1]. Obesity has also been proposed as a contributing factor [2]. Rather than forming a discrete mass, grossly it presents as diffuse enlargement of the salivary gland, typically occurring unilaterally and progressing slowly [3, 4].

Histopathologically, salivary gland lipomatosis is characterized by the infiltration of well‐differentiated adipocytes within the salivary gland, which separates individual serous lobules into varying sizes. Typically, the glandular cells show no pathologic changes. However, some inflammatory foci and secondary changes, such as acinar atropy and fibrosis, can be observed [1, 2, 5]. Additionally, although salivary gland lipomatosis is typically not associated with a distinct peripheral capsule, there have been reported cases in which capsule formations has been described [1]. Surgical excision is curative if complete margins are achieved. Local recurrence is not expected [1, 4].

This condition should be differentiated from other histologically similar lesions, such as infiltrative lipoma and sialolipoma [1, 4]. Infiltrative lipoma is a benign variant of adipose tumor that forms a discrete mass and infiltrates surrounding tissues, whereas lipomatosis is characterized by the absence of discrete mass formation. In this case, the adipocytes formed multiple lobules within the salivary gland, rather than a discrete mass. As for sialolipoma, the main histologic feature distinguishing it from lipomatosis is its clear demarcation, which was not observed in this case. Therefore, the histopathologic characteristics of the present case are consistent with salivary gland lipomatosis.

As the diagnosis of salivary gland lipomatosis primarily relies on histopathologic evaluation, its cytological characteristics have not been well described. To the best of the author's knowledge, this is the first cytological description of canine salivary gland lipomatosis. In cases where unilateral enlargement of the salivary gland is observed, and fine needle aspiration cytology shows a mixture of well‐differentiated adipocytes and clusters of salivary gland cells, salivary gland lipomatosis should be considered as a differential diagnosis.

## Conflicts of Interest

The authors declare no conflicts of interest.
